# Pediatrics

**Published:** 1905-12

**Authors:** Maud J. Frye

**Affiliations:** Buffalo


### Pediatrics

Conducted by MAUD J. FRYE, M. D., Buffalo.

After Effects of Diphtheria on the Heart. White (*Jour. Am. Med. Assn.* Oct. 21, '05) discusses the above subject, basing his article upon a study of nearly 1,000 cases of diphtheria treated at the Boston City Hospital. He concludes: 1. Cardiac disturbance after diphtheria usually presents the picture of a mitral insufficiency with irregular heart action and few symptoms. Occasional cases have rapid pulse or cardiac irregularity without any other signs. 2. Moderate disturbance of the heart is very common after diphtheria and in a large number of cases persists from two to six months after the original illness. 3. In many cases the cardiac lesion does not clear up in the first half year, but lasts much longer; some ultimately recover; others probably do not. The duration of the heart trouble is usually in proportion to the severity of the original illness. 4. The fact that children often have few heart symptoms after diphtheria must not mislead us as to the importance of the injury to the heart. 5. Cardiac disturbance of long duration following diphtheria may be entirely recovered from It is not necessary to give up hope of recovery in individual long cases. 6. The treatment of this condition consists in a sufficient period of rest in bed, and then in watching the effects of mild exercise on the heart for several months at least and grading it to meet individual requirements.

---

Treatment of Diarrhea in Children. Winters (*Med. News,* July 15, 1905) presents an excellent article from which the following points are taken: First castor oil is to be given: a teaspoonful up to 6 months; a dessertspoonful up to 1 year; a tablespoonful to a child a year and a half old. Give it ice cold. Give nothing two hours before and after giving the oil. If it is rejected, repeat in an hour. Sometimes opium aids the effect of oil in overcoming spasm. After the effect of the oil has ended for 24 hours or longer only water is to be given. In young infants hot water is preferable. With high temperature cold water is grateful. Heed the child's preference. Food may be witheld even 72 hours. Begin feeding with milk with the proteid contents reduced—(Dr. Winters condemns the use of dextrinised cereals.) For a baby of three months use one teaspoonful of one ounce of top cream from a quart bottle, mixed with one ounce of unboiled filtered water and 2 teaspoonfuls of lime water. Feed one ounce every 4 hours. Midway between feedings give 1-2 ounces of water. At the end of 24 hours if child is doing well drop the water and feed every 2 hours. In two days the child if doing well may be put on food made of 2 ounces of top cream, ½ ounce being added to 2 ounces of water and ½ ounce lime water for a feeding. Give every 3 hours, seven feedings in 24 hours. Gradually increase to normal strength.

For a child of six months, mix 2 teaspoonfuls of one ounce top cream with 1½ ounces cold, unboiled, filtered water, and 3 teaspoonfuls of lime water. Give 1½ ounces every 4 hours, alternating with 1½ to 2 ounces of water every 4 hours. At the end of 24 hours take ½ ounce of the top ounce of cream, mix with 3 ounces of water and ½ ounce of lime water. Feed 3 ounces every 3 hours and if baby is not satisfied, give water immediately after feeding, not between. The infant continuing to do well, in 48 hours prepare food by taking 2 ounces of top cream (only two ounces being taken from each bottle) mixed with 3½ ounces of water and one ounce of lime water. Feed 4 ounces every 3 hours, 7 feedings in 24 hours. In 3 days the food may be still further strengthened by using 1½ ounces of the top 4 ounces of cream, mixed with 3½ ounces of water and one ounce of lime water. Feed this quantity every 3½ hours, 6 feedings in 24 hours. Gradually increase the strength of the food to that proper for its age.

For children above one year the cereals are of greatest value. Those to be used are barley (whole or pearl barley) Imperial Granum, arrowroot, rice, cream of wheat. Cook with water; cooking arrowroot, and Imperial Granum ½ hour, pearl barley 3 hours, rice and cream of wheat one hour. Barley, rice and cream of wheat need straining. Do not give with cream, milk or sugar but from a bottle as a gruel or hot with a little butter and salt. Feed 4 ounces 5 times a day to a child a year old.

In neglected cases with a subacute or chronic diarrhea Dr. Winters uses either breast milk or condensed milk. To use condensed milk for a baby 3 mos. old take 1 level teaspoonful of sweetened condensed milk and add to it 24 teaspoonfuls of boiling water. Use one ounce for each feeding and prepare fresh each time. Add 2 teaspoonfuls of lime water to each feeding. Feed every 4 hours alternating with 1 ounce of water and 2 teaspoonfuls of lime water given every 4 hours. In 48 hours if the child is improving feed 2 ounces and double the amount of water and lime water given alternately. In another 48 hours, if the movements have become normal, give 2 ounces of the condensed milk mixture with ½ ounce of lime water every 2 hours and discontinue the water. In a week if everything is normal the food is strengthened by making it 1 part condensed milk to 16 of water and giving 3 ounces with ½ ounce of lime water every 3 hours. With the approach of cold weather, one teaspoonful of one ounce of top cream is added first to every other bottle, later to every bottle, then two teaspoonfuls, and gradually the condensed milk is withdrawn and top milk increased.

Dr. Winters regards irrigation as an abused treatment. To supplement the action of castor oil it may be expedient to irrigate on the first day.

[In Buffalo the use of unboiled water can hardly be advised.]

